# Serial inflammation imaging with pericoronary adipose tissue in patients with immunoglobulin G4-related coronary periarteritis: a case report

**DOI:** 10.1093/ehjcr/ytaf271

**Published:** 2025-05-28

**Authors:** Satoshi Kitahara, Yu Kataoka, Yusuke Fujino

**Affiliations:** Department of Cardiology, Kashiwa Kousei General Hospital, 617 Shikoda, Kashiwa 277-0862, Japan; Department of Cardiovascular Medicine, National Cerebral and Cardiovascular Centre, 6-1, Kishibe-Shinmachi, Suita, Osaka 564-8565, Japan; Department of Cardiovascular Medicine, National Cerebral and Cardiovascular Centre, 6-1, Kishibe-Shinmachi, Suita, Osaka 564-8565, Japan; Department of Cardiology, Kashiwa Kousei General Hospital, 617 Shikoda, Kashiwa 277-0862, Japan

**Keywords:** Pericoronary adipose tissue, Immunoglobulin G4-related disease, Inflammation, Case report

## Abstract

**Background:**

Immunoglobulin G4-related disease (IgG4-RD) is a systemic immune-mediated inflammatory disease that infrequently involves the coronary arteries. Given that pericoronary adipose tissue (PCAT) attenuation reflects the degree of inflammation in the coronary arteries, monitoring inflammation with PCAT may enable evaluation of disease activity in IgG4-related coronary periarteritis (CP).

**Case summary:**

A 58-year-old man with a history of IgG4-RD presented with ST-segment elevation myocardial infarction. Emergent coronary angiography revealed a severe stenotic lesion in the mid-segment of his left circumflex artery (LCX). Intravascular ultrasound (IVUS) imaging demonstrated thickening of the adventitia, and optical coherence tomography (OCT) showed the formation of vasa vasorum in the proximal segment of the LCX. Along with an elevated IgG4 level (1890 mg/dL), he was diagnosed with IgG4-related CP. Coronary computed tomography angiography (CCTA) after percutaneous coronary intervention (PCI) revealed soft tissue proliferation with elevated PCAT attenuation [PCAT_LCX_ attenuation = −68.4 Hounsfield units (HU)] around the proximal LCX. Following the initiation of prednisolone, the IgG4 level decreased to 239 mg/dL at 8 months post-PCI. Follow-up IVUS showed reduced adventitial thickness, and most of the previously observed vasa vasorum had disappeared on OCT. Furthermore, CCTA demonstrated a reduction in PCAT_LCX_ attenuation (to −81.8 HU), accompanied by a reduction in soft tissue volume.

**Discussion:**

In this case, serial PCAT analysis demonstrated resolution of inflammatory activity in response to prednisolone therapy. Serial PCAT imaging may have potential for evaluating disease activity and monitoring response to anti-inflammatory therapy in patients with IgG4-RD.

**Learning points**

- Immunoglobulin G4 (IgG4)-related coronary periarteritis (CP) is associated with inflammation.
- Pericoronary adipose tissue (PCAT) imaging could identify inflammatory activity of IgG4-related CP.
- Serial PCAT imaging may have a potential to evaluate disease activity and response to anti-inflammatory therapies in patients with IgG4-related CP.

## Introduction

Immunoglobulin G4-related disease (IgG4-RD) is a systemic immune-mediated inflammatory disease, which infrequently involves the coronary arteries. Given that pericoronary adipose tissue (PCAT) attenuation reflects the degree of inflammation in coronary arteries, monitoring inflammation with PCAT may enable evaluation of disease activity in IgG4-related coronary periarteritis (CP).

## Summary figure

**Figure:**
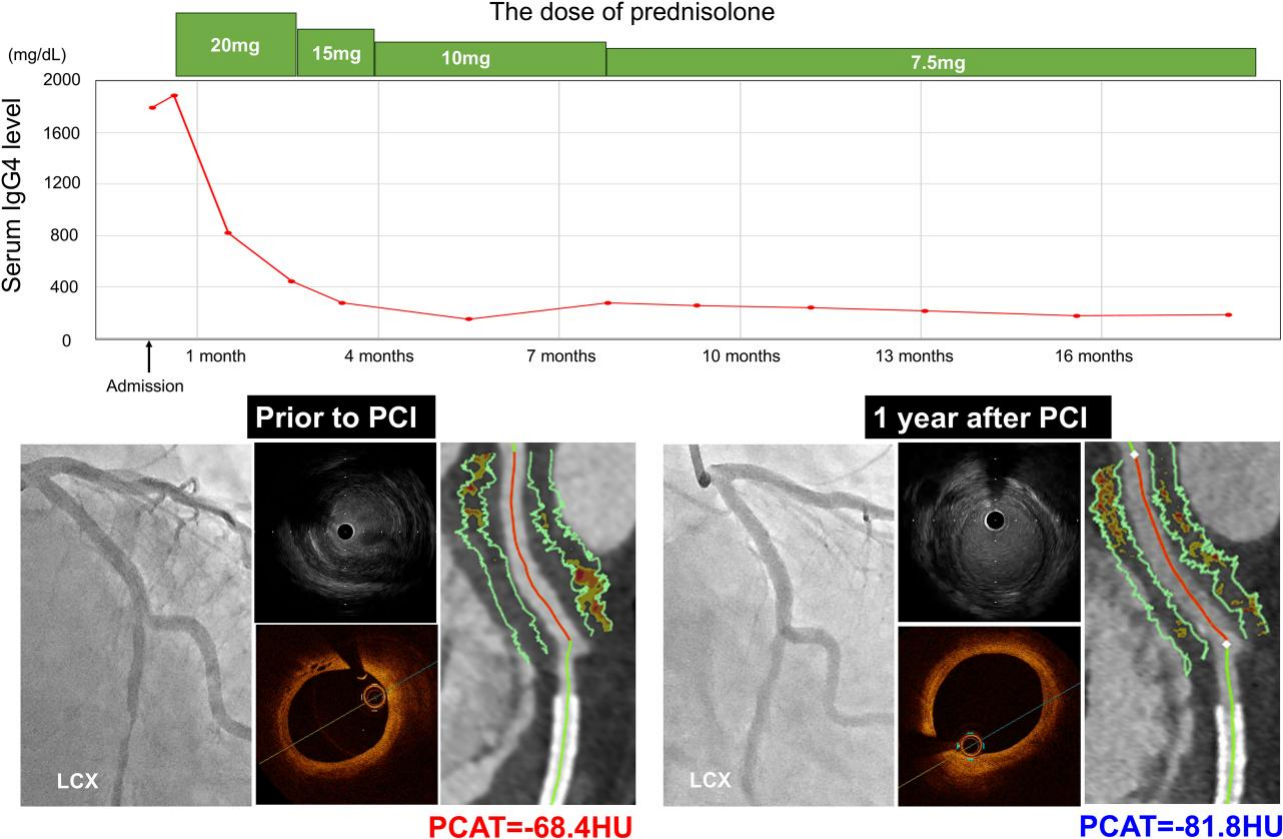


## Case presentation

A 58-year-old gentleman presented to the emergency department with syncope and prolonged back pain. He had a history of being an ex-smoker, as well as hypertension, Type 2 diabetes mellitus, and hyperuricaemia. He was already treated with 5 mg rosuvastatin, 40 mg nifedipine, 5 mg bisoprolol, 20 mg olmesartan, 25 mg spironolactone, and 10 mg febuxostat. In addition, he had a history of submandibular gland swelling at age 53, which led to the diagnosis of IgG4-RD. Following the diagnosis, 40 mg prednisolone had been commenced; however, he discontinued this therapy on his own at age 54.

At the emergency department, his initial blood pressure was 96/81 mmHg and heart rate was 90 b.p.m. There were no remarkable findings on physical examination, including cardiac murmurs or abnormal respiratory sounds. The electrocardiogram showed ST-segment elevation in leads II, III, aVF, and V5–6, with ST-segment depression in leads V1–3. Additionally, echocardiography revealed reduced wall motion in the infero-posterior region. While there was no evidence of elevated cardiac enzymes, an elevated IgG4 level (1796 mg/dL) was observed, accompanied by an elevated erythrocyte sedimentation rate (56 mm/h) and a high-normal level of C-reactive protein (0.16 mg/dL). He was diagnosed with ST-segment elevation myocardial infarction (STEMI), and emergent coronary angiography was conducted.

**Figure 1:**
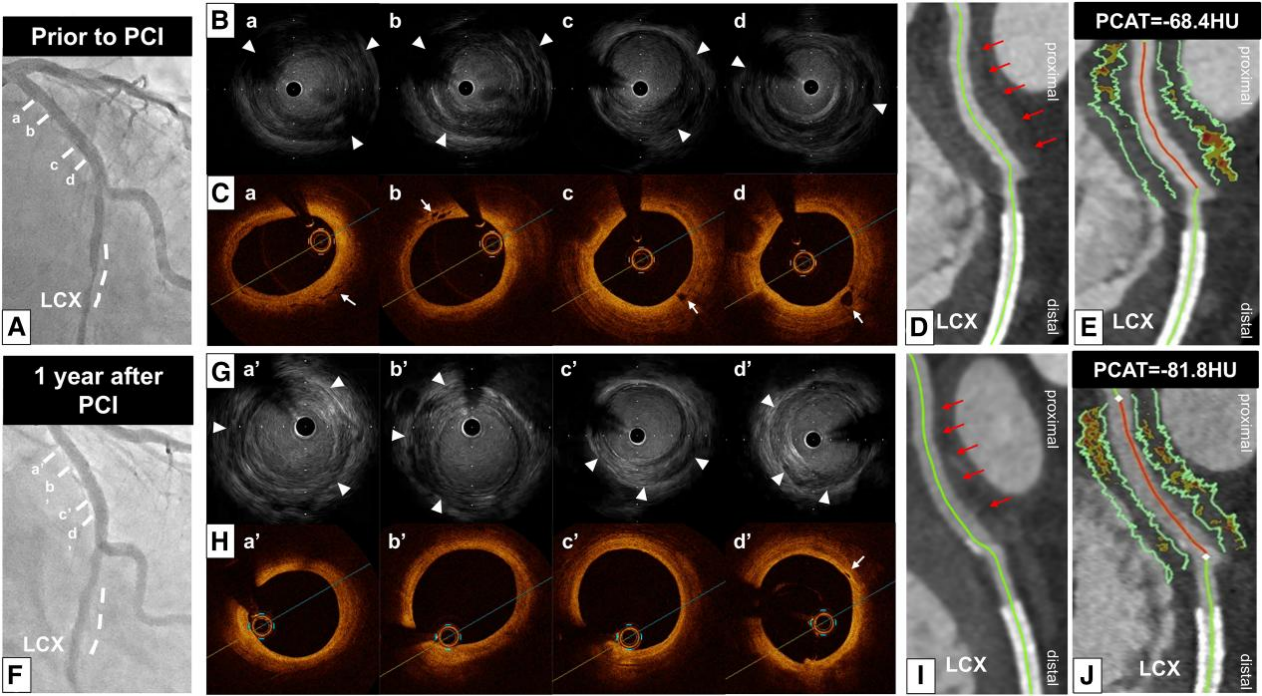
(*A*) Emergent coronary angiography demonstrated severe stenosis in the distal segment of the left circumflex artery (dotted white line). a–d correspond to the intravascular ultrasound and optical coherence tomography images in *B* and *C*. (*B*) Intravascular ultrasound imaging revealed thickening of the adventitia (white arrowheads) at the proximal segment of the left circumflex artery. (*C*) Optical coherence tomography imaging showed vasa vasorum at the proximal segment of the left circumflex artery (white arrows). (*D*) Coronary computed tomography angiography imaging 12 days after primary percutaneous coronary intervention demonstrated soft tissue proliferation surrounding the proximal segment of the left circumflex artery. (*E*) The average pericoronary adipose tissue attenuation at this segment was −68.4 HU. (*F*) Coronary angiography at 1 year following the commencement of prednisolone did not identify any progression of coronary artery stenosis. The dotted white line indicates the segment where a stent had previously been implanted. (*G*) A reduction in the thickness of the adventitia (white arrowheads) was observed on intravascular ultrasound imaging. (*H*) A reduction in the vasa vasorum was observed on optical coherence tomography imaging (white arrow). (*I*) Coronary computed tomography angiography imaging demonstrated a reduction in soft tissue volume. (*J*) Pericoronary adipose tissue attenuation decreased to −81.8 HU. IVUS, intravascular ultrasound; HU, Hounsfield units; LCX, left circumflex artery; OCT, optical coherence tomography; PCAT, pericoronary adipose tissue; PCI, percutaneous coronary intervention.

**Figure 2:**
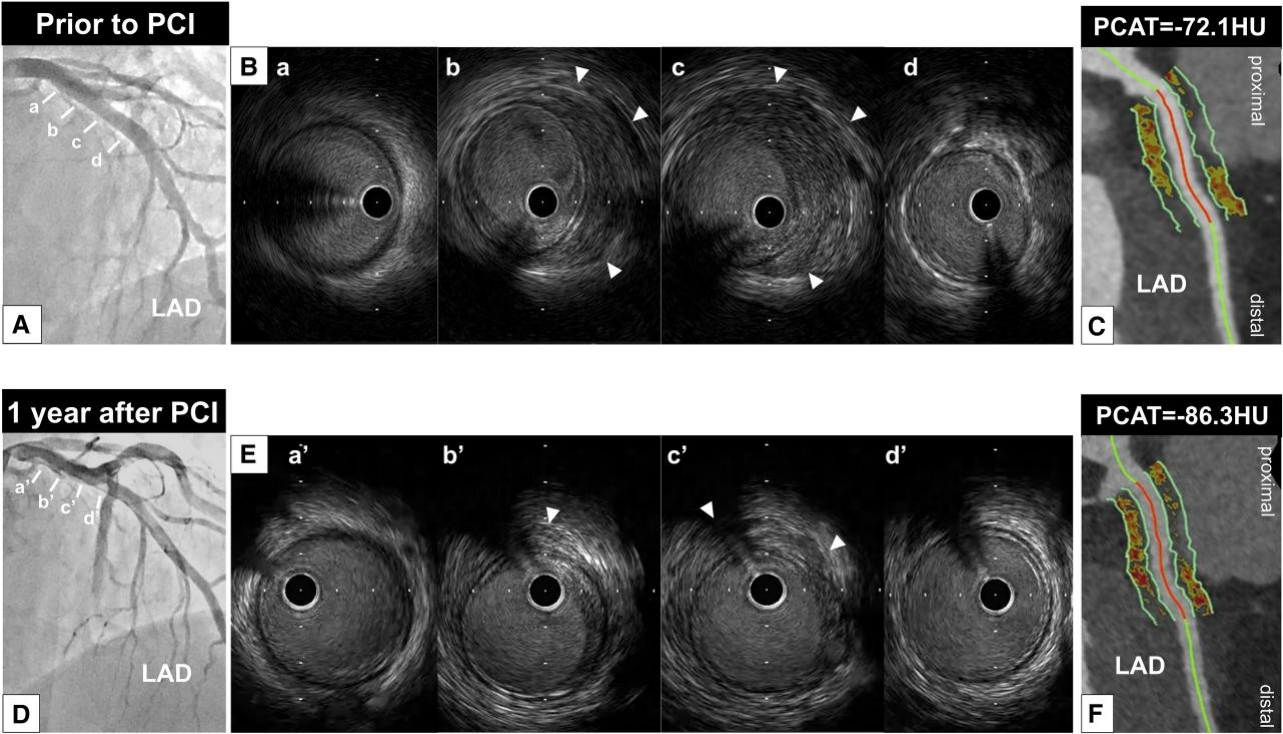
(*A*) No significant stenosis was observed in the left anterior descending artery. (*B*) Intravascular ultrasound imaging revealed thickening of the adventitia (white arrowheads) at the proximal segment of the left anterior descending artery. (*C*) Coronary computed tomography angiography imaging 12 days after primary percutaneous coronary intervention demonstrated soft tissue proliferation surrounding the proximal segment of the left anterior descending artery. The average pericoronary adipose tissue attenuation at this segment was −72.1 HU. (*D*) Coronary angiographic features remained unchanged 1 year after therapy. (*E*) A decrease in the thickness of the adventitia (white arrowheads) was observed on intravascular ultrasound imaging. (*F*) Coronary computed tomography angiography imaging demonstrated a reduction in soft tissue volume, and pericoronary adipose tissue attenuation decreased to −86.3 HU. HU, Hounsfield units; IVUS, intravascular ultrasound; LAD, left anterior descending artery; PCAT, pericoronary adipose tissue attenuation; PCI, percutaneous coronary intervention.

**Figure 3:**
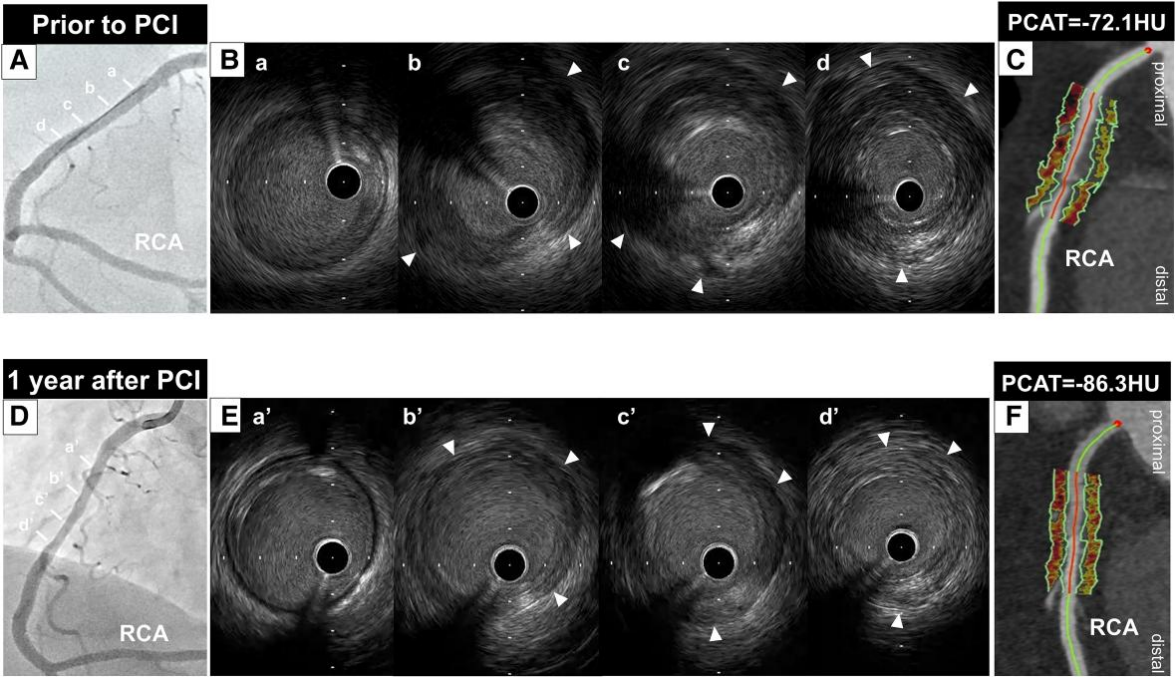
(*A*) Mild stenosis was identified in the proximal segment of the right coronary artery. (*B*) Intravascular ultrasound imaging revealed thickening of the adventitia (white arrowheads) at the proximal segment of the right coronary artery. (*C*) Coronary computed tomography angiography imaging 12 days after primary percutaneous coronary intervention demonstrated soft tissue proliferation surrounding the proximal segment of the right coronary artery. The average pericoronary adipose tissue attenuation at this segment was −87.1 HU. (*D*) Coronary angiography at 1 year after therapy did not show any progression of coronary artery stenosis. (*E*) A decrease in the thickness of the adventitia (white arrowheads) was observed on intravascular ultrasound imaging. (*F*) Coronary computed tomography angiography imaging demonstrated a reduction in soft tissue volume, and pericoronary adipose tissue attenuation decreased to −95.8 HU. HU, Hounsfield units; IVUS, intravascular ultrasound; PCAT, pericoronary adipose tissue attenuation; PCI, percutaneous coronary intervention; RCA, right coronary artery.

Coronary angiography identified a severe stenosis in the distal segment of the left circumflex artery (LCX) (*Figure 1A*) (see Supplementary material online, *Movie S1*). There was no significant stenosis in the left anterior descending artery (LAD) (*Figure 2A*), whereas mild stenosis was observed in the proximal segment of the right coronary artery (RCA) (*Figure 3A*). Intravascular ultrasound (IVUS) (AltaView™, Terumo, Tokyo, Japan) imaging prior to primary percutaneous coronary intervention (PCI) revealed a large amount of low-echoic plaque at the culprit lesion (see Supplementary material online, *Movie S2*).

Furthermore, thickening of the adventitia at both the culprit and its proximal adjacent segments was identified (*Figure 1B*; Supplementary material online, *Movie S2*). Notably, these features were similarly observed in both the LAD and RCA (*Figures 2B* and *3B*; Supplementary material online, *Movies S3* and *S4*).

Following completion of the primary PCI with implantation of one drug-eluting stent, we conducted optical coherence tomography (OCT) imaging to evaluate the adjacent segment of the culprit lesion in the LCX. Optical coherence tomography imaging demonstrated the formation of vasa vasorum in the proximal segment of the LCX (*Figure 1C*) (see Supplementary material online, *Movie S5*). Based on these intravascular imaging findings, the case was diagnosed as IgG4-related CP. The maximum creatine kinase level was 2384 U/L.

Further evaluation to assess the extent of CP was performed using coronary computed tomography angiography (CCTA) 12 days after PCI. Coronary computed tomography angiography was conducted using a fifth-generation dual-source CT scanner (SOMATOM Force; Siemens Healthcare, Forchheim, Germany) with a tube voltage of 90 kV. Using automated tube current modulation (CARE Dose4D, Siemens), the tube current was set at 410 mA. The corresponding segment in the proximal LCX exhibited soft tissue proliferation (*Figure 1D*). We also analysed PCAT, which reflects coronary artery inflammation, using semi-automated software (Aquarius 3D Workstation, TeraRecon Inc., San Mateo, CA, USA). Pericoronary adipose tissue attenuation was automatically quantified as the mean attenuation of all voxels within 3D concentric layers extending outward 3 mm from the operator-traced vessel wall.^1^ A substantially high PCAT attenuation [PCAT_LCX_ attenuation = −68.4 Hounsfield units (HU)] around the proximal LCX was visualized (*Figure 1E*). Soft tissue proliferation was also present in both the LAD and RCA, with PCAT_LAD_ and PCAT_RCA_ attenuation values of −72.1 HU and −87.1 HU, respectively (*Figures 2C* and *3C*). Thus, PCAT attenuation in this case was highest in the culprit vessel.

Following PCAT evaluation with CCTA, 20 mg prednisolone was initiated and then gradually tapered to 7.5 mg at 8 months post-PCI. After starting prednisolone, the IgG4 level decreased to 239 mg/dL, accompanied by a decrease in CRP (<0.01 mg/dL). As chest pain recurred 1 year after PCI, coronary arteries were re-evaluated via coronary angiography and multi-modality imaging (IVUS, OCT, and CCTA). No in-stent restenosis or disease progression was observed (*Figures 1F*, *2D*, and *3D*; Supplementary material online, *Movie S6*). Intravascular ultrasound imaging revealed decreased adventitial thickness in all imaged coronary arteries (*Figures 1G*, *2E*, and *3E*; Supplementary material online, *Movies S7–S9*). Additionally, OCT imaging showed that most of the previously identified vasa vasorum had disappeared (*Figure 1H*; Supplementary material online, *Movie S10*). Coronary computed tomography angiography was performed on the same dual-source CT scanner under nearly identical conditions (tube voltage: 90 kV; tube current: 483 mA) to avoid the influence of voltage changes.^2^ Interestingly, CCTA analysis visualized a reduction in PCAT attenuation across all imaged coronary arteries, accompanied by a reduction in soft tissue volume (*Figures 1I* and *J*, *2F*, and *3F*). His chest pain resolved spontaneously, and he has remained event free for 1.5 years after PCI while continuing on 7.5 mg prednisolone.

## Discussion

Immunoglobulin G4-related disease is a systemic immune-mediated inflammatory disease that infrequently involves the coronary arteries.^3^ Immunoglobulin G4-related CP is characterized by the formation of coronary aneurysms or multiple ectasias and stenoses in the coronary arteries. In addition, patients with IgG4-related CP have been shown to have an elevated risk of coronary artery events compared with those without CP.^4^ These observations suggest the importance of evaluating the coronary arteries in the setting of IgG4-RD.

Pathophysiologically, arteritis due to IgG4-RD is caused by inflammation and fibrosis.^3^ Given that PCAT attenuation reflects the degree of inflammation in coronary arteries, monitoring inflammation with PCAT may enable evaluation of disease activity in IgG4-related CP. Although IgG4-RD is generally responsive to steroid therapy, some patients are refractory to glucocorticoids and other immunosuppressive treatments. In our case, all three major coronary arteries were involved in IgG4-related CP, and the highest inflammatory activity on PCAT was observed in the proximal segment of the culprit vessel. Of note, serial PCAT analysis demonstrated a healing of inflammatory activity in response to the initiation of prednisolone. These favourable changes in inflammatory activity were accompanied by reductions in soft tissue proliferation and vasa vasorum.

To date, there are no studies that have investigated the efficacy of steroid treatment on coronary lesions in patients with IgG4-RD exist. A serial IVUS imaging study reported CRP as an independent contributor to plaque progression.^5^ It could be speculated that lowering CRP with steroid treatment might induce disease regression in patients with IgG4-RD.

Since our case had a history of smoking, hypertension, and diabetes mellitus, STEMI may have occurred due to these atherogenic risk factors. However, thickening of the adventitia was observed at the culprit site. Adventitial thickening has been shown to be one of the morphological characteristics of coronary lesions in patients with IgG4-RD.^6^ Therefore, we speculated that STEMI might be attributable to IgG4-RD.

In our case, we observed that IgG4-related CP responded favourably to steroid therapy, as shown through serial PCAT and intravascular imaging. Serial PCAT imaging may have potential for evaluating disease activity and response to anti-inflammatory therapies in patients with IgG4-RD.

## Supplementary Material

ytaf271_Supplementary_Data

## Data Availability

The data underlying this article will be shared on reasonable request to the corresponding author.

## Lead author biography

Satoshi Kitahara was born in Tokyo, Japan, in 1987. He graduated from Kumamoto University and received the PhD degree in 2023. He experienced a large number of PCI cases as well as analysis of intravascular imaging and coronary computed tomography angiography imaging. From 2022, he serves as a staff cardiologist at Kashiwa Kousei General Hospital, Chiba, Japan. His research interests are percutaneous coronary intervention, intravascular imaging, coronary computed tomography angiography imaging, and lipids.

## Supplementary material

**Consent:** The authors confirm that written consent for submission and publication of this case report including the images and associated text has been obtained from the patient in line with COPE guidance.

**Funding:** There is no funding support.
